# Real world data on outcomes of anti-CD38 antibody treated, including triple class refractory, patients with multiple myeloma: a multi-institutional report from the Canadian Myeloma Research Group (CMRG) Database

**DOI:** 10.1038/s41408-023-00946-z

**Published:** 2023-12-08

**Authors:** A. Visram, A. De La Torre, D. White, J. Su, E. Masih-Khan, M. Chu, V. Jimenez-Zepeda, A. McCurdy, R. LeBlanc, K. Song, H. Mian, M. Louzada, M. Sebag, D. Bergstrom, J. Stakiw, A. Reiman, R. Kotb, M. Aslam, C. Venner, R. Kaedbey, E. Gul, D. Reece

**Affiliations:** 1 Department of Medicine, The Ottawa Hospital, The Ottawa Hospital Research Institute, Ottawa, ON Canada; 2 Division of Hematology, Dalhousie University and Queen Elizabeth II Health Sciences Centre, Halifax, NS Canada; 3 Canadian Myeloma Research Group, Toronto, ON Canada; 4 Department of Medical Oncology and Hematology, Princess Margaret Cancer Centre, Toronto, ON Canada; 5 Department of Oncology, Cross Cancer Institute, Edmonton, Alberta, Edmonton, AB Canada; 6 Tom Baker Cancer Center, Department of Hematology, University of Calgary, Calgary, AB Canada; 7 Hôpital Maisonneuve-Rosemont, Université de Montréal, Montreal, QC Canada; 8 The Leukemia/Bone Marrow Transplant Program of BC, British Columbia Cancer Agency, Vancouver, Canada; 9 Juravinski Cancer Centre (Hamilton-CCO), Hamilton, ON Canada; 10 University of Western Ontario, London Health Sciences Centre, London, ON Canada; 11 Division of Hematology, McGill University Health Centre, Montreal, QC Canada; 12 Division of Hematology, Memorial University of Newfoundland, St John’s, Newfoundland and Labrador, Canada; 13 Saskatoon Cancer Centre, Saskatoon, SK Canada; 14 Oncology, Saint John Regional Hospital, Saint John, NB Canada; 15 Medical Oncology and Hematology, Cancer Care Manitoba, Winnipeg, MB Canada; 16 Allan Blair Cancer Center, Regina, SK Canada; 17 BC Cancer – Vancouver Centre, Lymphoma and Myeloma Program, University of British Columbia, Vancouver, BC Canada; 18 Segal Cancer Centre, Jewish General Hospital, McGill University, Montreal, Montreal, QC Canada

**Keywords:** Epidemiology, Myeloma, Cancer epidemiology, Myeloma

## Abstract

Multiple myeloma (MM) remains incurable despite the availability of novel agents. This multi-center retrospective cohort study used the Canadian Myeloma Research Group Database to describe real-world outcomes of patients withanti-CD38 monoclonal antibody (mAb) refractory MM subsequently treated with standard of care (SoC) regimens. Patients with triple class refractory (TCR) disease (refractory to a proteasome inhibitor, immunomodulatory drug, and anti-CD38 mAb) were examined as a distinct cohort. Overall, 663 patients had disease progression on anti-CD38 mAb therapy, 466 received further treatment (346 with SoC regimens were included, 120 with investigational agents on clinical trial and were excluded). The median age at initiation of subsequent SoC therapy of 67.9 (range 39.6–89.6) years with a median of 3 prior lines (range 1–9). The median PFS and OS from the start of subsequent therapy was 4.6 (95% CI 4.1–5.6) months and 13.3 (95% CI 10.6–16.6) months, respectively. The median PFS and OS of patients with TCR disease (*n* = 199) was 4.4 (95% CI 3.6–5.3) months and 10.5 (95% CI 8.5–13.8) months. Our results reinforce that real-world patients with relapsed MM, particularly those with TCR disease, have dismal outcomes. There remains an urgent unmet need for the development of and access to effective therapeutics for these patients.

## Introduction

Multiple myeloma (MM) is a hematologic cancer arising from malignant plasma cells. It is the second most common hematologic malignancy with an age-standardized incidence rate of 8.5 cases per 100,000 Canadians in 2022 [1]. Over the past 20 years, the regulatory approval of several novel agents to treat MM has prolonged median patient survival from 3 to 8–10 years [2, 3]. However, despite the introduction of novel therapeutic agents in the upfront and relapsed treatment settings, MM remains incurable [4].

Long-term disease control requires ongoing access to effective therapeutic options at relapse. Recent therapeutics targeting the B-cell maturation antigen (BCMA) have shown promising efficacy in refractory patient populations; the United States Food and Drug Administration has recently approved a number of anti-BCMA therapies (idecabtagene vicleucel, ciltacabtagene autoleucel, teclistamab, and elranatamab) based on early phase studies demonstrating overall response rates (ORR) ranging from 63 to 95% in patients with >3 lines of therapy and prior treatment with a proteasome inhibitor (PI), an immunomodulatory agent (IMiD), and an anti-CD38 monoclonal antibody (mAb) [5–8]. However, the trials leading to FDA approval were single-arm studies. Outcomes of real-world patients with refractory MM treated with standard-of-care therapies are needed to serve as a benchmark and contextualize improvements in health outcomes with these emerging therapies.

A multicenter retrospective study performed in academic centers in the United States reported a median progression-free survival (PFS) and overall survival (OS), among patients with disease refractory to anti-CD38 mAbsthat received subsequent therapy, of 3.4 and 9.3 months, respectively [9]. However, given differences in drug accessibility with our publicly funded healthcare model, there is a need to understand the real-world outcomes of patients with MM refractory to anti-CD38 mAbs, particularly those with triple class refractory (TCR) disease, outside of the United States. Therefore, we performed a retrospective cohort study to describe the clinical characteristics, progression-free survival (PFS), and overall survival (OS) of patients treated for MM refractory to anti-CD38 mAb therapy. Outcomes of patients with TCR MM were examined as a distinct cohort. Given that both the Food and Drug Administration and European Medicines Agency have approved the use of some immune therapies for MM in patients who are triple class exposed (TCE) and not necessarily TCR, we also compared PFS and OS outcomes among patients with TCE versus TCR MM.

## Methods

### Patient population

Patients were identified using the Canadian Myeloma Research Group Database (CMRG-DB), a prospectively maintained national database comprising of more than 8700 patients diagnosed with MM since 2007 across 17 academic centers [10]. Included patients had MM that was refractory to an anti-CD38 mAb-based index regimen after at least 4 weeks of treatment and were subsequently treated with standard of care (SoC) regimens. At the time of data cutoff, anti-CD38 mAb-based regimens were not reimbursed in Canada in the frontline setting, and so the vast majority of patients received antiCD38 mAb regimens at relapse. To be considered refractory, patients had to have a progression of MM on therapy or within 60 days of the last dose of the anti-CD38 mAb-containing regimen, as defined by the International Myeloma Working Group Response Criteria [11]. Patients were excluded if they discontinued anti-CD38 mAb therapy for reasons other than progressive disease, were treated with an anti-CD38 mAb for a plasma cell disorder other than MM, were palliated or lost to follow-up after anti-CD38 mAb progression, or were treated on a clinical trial with an investigational agent in the subsequent line of therapy after anti-CD38 mAb progression. The data cutoff date was June 30, 2022.

### Study definitions

The index regimen is defined as the anti-CD38 mAb-containing regimen. SoC treatments used subsequently after progression on the index regimen included IMiDs, PIs, anti-CD38 mAbs, anthracyclines, alkylating agents, and steroids. Patients with TCR MM were examined as a distinct cohort and were defined as patients refractory to an IMiD, PI, and an anti-CD38 mAb. Similarly, penta-refractory disease was defined as progression on treatment or within 60 days of the last dose of 1 CD38 mAb, 2 PI’s, and 2 IMiDs. Daratumumab, bortezomib, and dexamethasone (DVd) is a common treatment regimens; however, bortezomib is only reimbursed for a total of 8 cycles in some provinces (in keeping with the CASTOR trial protocol [12]), whereas other provinces continue bortezomib until progression. Therefore, the dates of the last bortezomib treatment were ascertained for patients on DVd, to clarify whether patients were in fact bortezomib-refractory at progression on in the index regimen. Patients with MM not meeting TCR criteria were defined as being TCE if they had been previously treated with an IMiD, PI, and an anti-CD38 mAb.

Response rates were determined as per modified IMWG guidelines, wherein complete response (CR) was defined as the absence of a monoclonal protein on serum protein electrophoresis, serum immunofixation (IFE), and urine immunofixation even if a bone marrow was not performed to confirm response [13]. The overall response rate (ORR) was defined as a partial response (PR) or better [13]. High-risk cytogenetics was defined as the presence of t(4;14), t(14;16) or del(17p)—given that gain1q/amp1q were not routinely tested across Canada during the study timeframe, this data was omitted. PFS was defined as the time between initiation of subsequent therapy after progression on the index regimen until next progression (as defined by the IMWG criteria [13]) or death. OS was defined as the time between initiation of subsequent therapy and death or date of the last known follow-up.

### Statistical analysis

Time-to-event analyses were used to determine the PFS and OS. Survival curves were constructed using the Kaplan–Meier method, and the impact of covariates of interest were assessed using the log rank test. Multivariable analysis was used to assess the impact of various risk factors on OS and PFS. Variables included in the PFS and OS analyses included age at time of progression on index regimen (≥75 versus <75 years), high-risk status at diagnosis (yes versus no versus unknown), sex (male versus female), depth of response on index regimen (<VGPR versus ≥VGPR), number of prior treatment lines (≥3 prior lines including the index regimen versus <3 prior lines), time from diagnosis to progression on the index regimen (≥4 years versus <4 years). A stepwise forward selection process was used to select the covariates included in forest plot multivariable analyses; only covariates with a *p* value < 0.05 were included. HR were reported with 95% confidence intervals. The fit of the final model was verified with the Hosmer and Lemeshow test, and collinearity between independent variables was verified by correlation analysis, for variables with strong collinearity (correlation coefficient > 0.9), one of the two variables was excluded from the multivariable analysis according to the biological plausibility. Confounding was assessed by monitoring the changes in the model parameters when adding new variables. If substantial changes (i.e. >20%) were observed in the regression coefficients, this was considered as indicative of confounding. The variables were considered as factors of achieving an overall response (≥PR) when the odds ratio (OR) was greater than 1.0 and the *p* value was less than or equal to 0.05.

Ethics approval for this study was obtained from the Ottawa Hospital Institutional Review Board. All patients provided written or implied informed consent. The study was conducted in accordance with the Declaration of Helsinki.

## Results

### Patient characteristics

The CMRG-DB identified 663 patients with MM refractory to anti-CD38 mAb therapy. From them, 466 (466/663, 70%) patients initiated a subsequent regimen, 145 (145/663, 22%) pursued palliative care, and 52 (52/663, 8%) were lost to follow-up. Of the 197 patients not receiving subsequent treatment at the time of progression or lost to follow-up, the median age was 72 years, and the median overall survival from the time of index regimen progression was 1.3 (95% CI 0.4–1.7) months. Of these untreated patients, 137 (137/197, 70%) had TCR MM, and their median age at progression on the index regimen was 71 years (range 42–91) with a median of 4 (range 2–9) lines of treatment including the index regimen. Overall, 42 patients (42/663, 6.3%) were had penta-refractory MM at progression on the index regimen; 22 did not undergo further treatment and 20 received subsequent treatment on a clinical trial (*n* = 11) or with SoC regimens (*n* = 9).

### Treatment post CD38-mAb progression

**Fig. 1:**
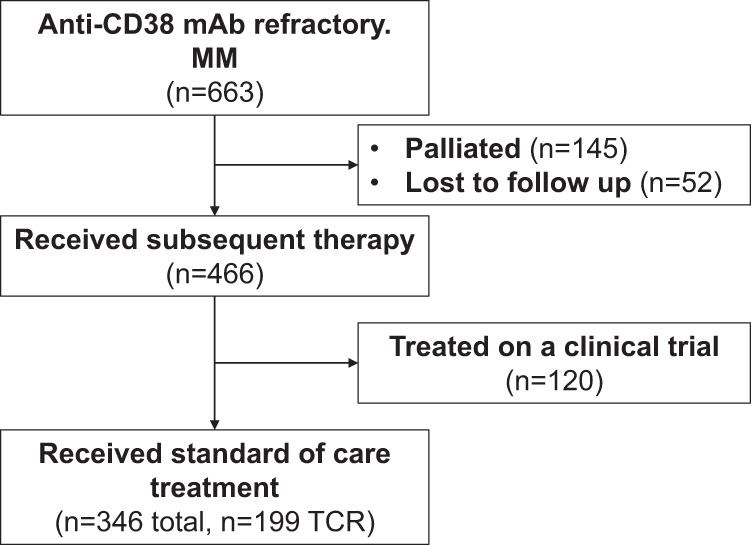
Consort diagram of included patients. Abbreviations: monoclonal antibody (mAb) triple class refractory (TCR).

**Fig. 2:**
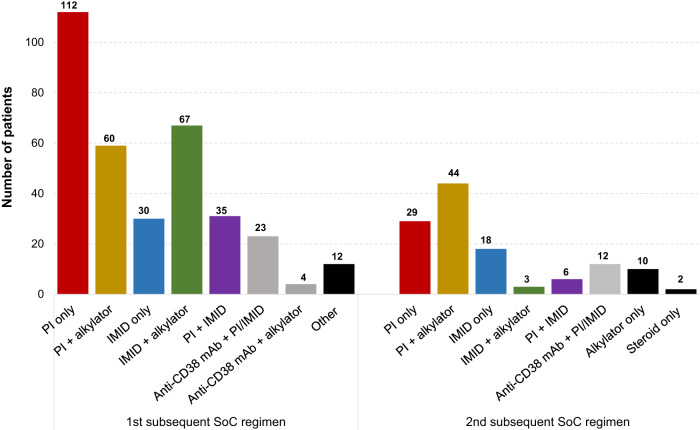
Frequency of standard of care (SoC) drug regimens used after relapse on the index anti-CD38 monoclonal antibody containing regimen. All regimens were given in conjunction with steroids. Of the patients with MM relapsing on 1st SoC therapy after index progression, 130 were treated with a 2nd Soc therapy and 81 were treated on clinical trial.

Of the 466 patients receiving subsequent treatment, 120 patients were treated on clinical trial and were excluded. Therefore, 346 patients treated with SoC regimens were included in this study, as shown in Fig. 1. Most patients (338/346, 98%) had MM refractory to daratumumab, with only a minority (8/346, 2%) receiving isatuximab as part of the index regimen. Seven patients progressed on a first-line regimen containing daratumumab. Sixty-three percent (218/346) of included patients previously received an autologous stem cell transplant, and most patients had MM refractory to lenalidomide or bortezomib (305/346 or 88% and 184/346 or 53%, respectively), as shown in Table 1. Of the 109 patients with disease progression on DVd, 87 had bortezomib (and TCR) refractory disease and 22 had non-TCR MM at progression (18 patients had disease progression on bortezomib, and 4 patients discontinued bortezomib >60 days prior to progression on DVd). The median age at initiation of subsequent therapy was 68 (range 40–90) years. The median time from diagnosis to initiation of subsequent SoC therapy after progression on the index regimen was 57 (range 6–283) months, and the median number of prior treatment lines was 3 (range 1–9). The most common SoC regimen used after progression on the index regimen was a PI/steroid doublet, followed by either a combination of PI or IMiDs with an alkylator (most commonly cyclophosphamide), as summarized in Fig. 2 and Table 2. PFS and OS outcomes, stratified by the most common regimens, are shown in Fig. S1. Twenty-seven patients (27/346, 8%) were re-treated with a CD38-mAb at progression on the index regimen (25 of these patients switched to a different anti-CD38 mAb), with a median washout period of 1.1 (range 0–7.0) months.

**Table 1:** Characteristics of patients with anti-CD38 mAbs refractory MM that were treated with subsequent standard of care therapy.

	Non-TCR	All TCR	Total
	(*n* = 147)	(*n* = 199)	(*n* = 346)
Median age at initiation of subsequent regimen—years (range)	68.4 (40.6–87.3)	67.4 (39.6–89.6)	67.9 (39.6–89.6)
Male sex—*n* (%)	85 (58)	108 (54)	193 (56)
Cytogenetic risk at diagnosis—*n* (%)			
High-risk^a^	37 (25)	48 (24)	85 (25)
Standard-risk	80 (54)	98 (49)	178 (51)
NA	30 (20)	53 (27)	82 (24)
ISS Stage at diagnosis—*n* (%)			
Stage I	31 (21)	42 (21)	73 (21)
Stage II	48 (33)	56 (28)	104 (30)
Stage III	45 (31)	58 (29)	103 (30)
Unknown	23 (16)	43 (22)	66 (19)
Median time from diagnosis to progression on index regimen—months (range)	57 (6–283)	50 (3–236)	52 (3–283)
Median number of lines of therapy at time of progression (inclusive of index regimen)—*n* (range)	2 (1–9)	3 (2–9)	3 (1–9)
Exposure to prior therapy—*n* (%)			
ASCT	96 (65)	122 (61)	218 (63)
Lenalidomide	134 (91)	199 (100)	333 (96)
Pomalidomide	38 (26)	45 (23)	83 (24)
Thalidomide	10 (7)	5 (3)	15 (4)
Bortezomib	129 (88)	192 (97)	321 (93)
Carfilzomib	6 (4)	38 (19)	44 (13)
Ixazomib	5 (3)	36 (18)	41 (12)
Refractory to prior therapy—*n* (%)			
Lenalidomide	109 (74)	196 (99)	305 (88)
Pomalidomide	34 (23)	42 (21)	76 (22)
Thalidomide	1 (1)	2 (1)	3 (1)
Bortezomib	22 (15)	162 (81)	184 (53)
Carfilzomib	0 (0)	33 (17)	33 (10)
Ixazomib	0 (0)	33 (17)	33 (10)

Percentages may not add to 100 due to rounding.

*TCR* triple class refractory, *ASCT* autologous stem cell transplant.

^a^High risk cytogenetics was defined as the presence of t(4;14), t(14;16), or del(17p).

**Table 2:** Characteristics of patients with anti-CD38 mAb refractory MM stratified by the subsequent standard of care therapy received after progression on the index regimen.

	KD	PCD	KCD	PD	Other regimens	Total
	(*N* = 100)	(*N* = 57)	(*N* = 33)	(*N* = 23)	(*N* = 133)	(*N* = 346)
Median age at initiation of subsequent regimen - years (range)	67 (41–84)	69 (46–88)	70 (46–87)	77 (57–90)	66 (40–87)	68 (40–90)
Male sex—n (%)	59 (59)	30 (53)	16 (49)	12 (52)	76 (57)	193 (56)
Cytogenetic risk at diagnosis—*n* (%)						
High-risk^a^	18 (18)	13 (23)	12 (36)	5 (22)	36 (27)	84 (24)
Standard-risk	52 (52)	15 (26)	11 (33)	7 (30)	40 (30)	125 (36)
Missing	18 (18)	13 (23)	12 (36)	5 (22)	57 (43)	137 (40)
Median number of lines of therapy at progression (inclusive of index regimen)—*n* (range)	2 (2–9)	3 (2–6)	3 (2–8)	3 (2–7)	3 (1–9)	3 (1–9)
Exposure status at progression on index regimen—*n* (%)						
ASCT	69 (69)	37 (65)	20 (61)	10 (44)	82 (62)	218 (63)
Lenalidomide	99 (99)	51 (90)	31 (94)	23 (100)	129 (97)	333 (96)
Pomalidomide	23 (23)	1 (2)	11 (33)	1 (4)	47 (35)	83 (24)
Thalidomide	4 (4)	1 (2)	3 (9)	0 (0)	7 (5)	15 (4)
Bortezomib	95 (95)	57 (100)	31 (94)	23 (100)	115 (87)	321 (93)
Carfilzomib	4 (4)	11 (19)	1 (3)	3 (13)	25 (19)	44 (13)
Ixazomib	15 (15)	6 (11)	1 (3)	3 (13)	16 (12)	41 (12)
Refractory status at progression on index regimen—*n* (%)						
Lenalidomide	95 (95)	44 (77)	29 (88)	22 (96)	115 (87)	305 (88)
Pomalidomide	19 (19)	1 (2)	10 (30)	1 (4)	45 (34)	76 (22)
Thalidomide	1 (1)	0 (0)	0 (0)	0 (0)	2 (2)	3 (1)
Bortezomib	39 (39)	47 (83)	18 (54.5%)	16 (70)	64 (48)	184 (53)
Carfilzomib	3 (3)	8 (14)	0 (0%)	2 (9)	20 (15)	33 (10)
Ixazomib	11 (11)	4 (7)	1 (3.0%)	3 (13)	14 (11)	33 (10)
Response rates—*n* (%)						
PD	15 (18)	10 (23)	5 (20)	3 (19)	36 (34)	69 (25)
SD	27 (33)	5 (11)	7 (28)	5 (31)	18 (17)	62 (23)
MR	1 (1)	3 (7)	1 (4)	1 (6)	5 (5)	11 (4)
PR	18 (22)	11 (25)	5 (20)	2 (13)	24 (23)	60 (22)
VGPR	17 (21)	9 (21)	6 (24)	4 (25)	17 (16)	53 (19)
CR/nCR	4 (5)	6 (14)	1 (4)	1 (6)	6 (6)	18 (7)
Not evaluable	18	13	8	7	27	73
ORR	39 (48)	26 (59)	12 (48)	7 (44)	47 (44)	131 (48)
Median PFS—months (95% CI)	4.3 (3.4, 6.0)	6.4 (3.7, 9.2)	6.2 (4.6, 9.3)	3.6 (2.1, 6.3)	4.2 (3.0, 5.3)	4.6 (4.1, 5.6)
Median OS—months (95% CI)	11.6 (9.0, 18.2)	11.1 (8.0, 22.0)	14.2 (7.9, NR)	7.6 (3.8, 21.7)	13.4 (9.6, 17.4)	13.3 (10.6, 16.6)

^a^High-risk cytogenetics was defined as the presence of t(4;14), t(14;16), or del(17p).

*KD* carfilzomib and dexamethasone, *PCD* pomalidomide and cyclophosphamide and dexamethasone, *KCD* carfilzomib and dexamethasone and cyclophosphamide, *PD* pomalidomide and dexamethasone, *ASCT* autologous stem cell transplant, *PFS* progression-free survival, *OS* overall survival.

### Efficacy outcomes post CD38-mAb progression

**Fig. 3:**
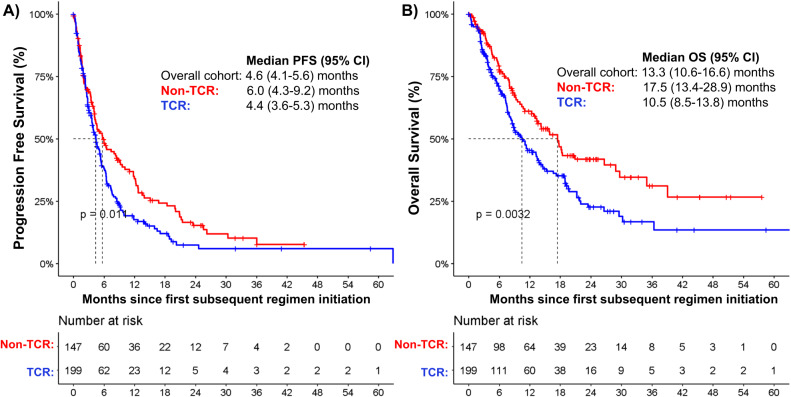
Outcomes of patients with TCR versus non-TCR RRMM. **A** Progression-free survival (PFS) and **B** Overall survival (OS) from initiation of subsequent standard of care therapy post anti-CD38 mAb progression.

The median follow-up from the date of initiation of subsequent SoC therapy was 8 months. Among the entire cohort of patients with MM refractory to an anti-CD38 mAb and treated with subsequent SoC therapy (*n* = 346), the median PFS from start of subsequent therapy was 4.6 (95% CI 4.1–5.6) months, and the median OS was 13.3 (95% CI 10.6–16.6) months (Fig. 3). The median PFS and OS was similar after excluding the 7 patients that had disease relapse on front-line daratumumab (median PFS 4.6 [95% CI 4.0–5.5] months, median OS 13.2 [95% CI 10.4–15.3] months). The overall response rate (ORR) to first subsequent SoC therapy was 48% (131/273), and 7% (18/273) achieved at least a complete response (CR) among patients evaluable for response assessment, as shown in Table 3. Among the 84 patients with high-risk cytogenetic markers at diagnosis, the median PFS and OS from initiation of subsequent therapy after progression on the index regimen were 3.5 (95% CI 2.5–6.3) months and 10.7 (95% CI 7.2–14.2) months, respectively. Response rates for high-risk patients treated with SoC subsequent therapy are summarized in Table S2.

**Table 3:** Best response (among evaluable patients) to subsequent SoC therapy after progression on the index regimen.

	Non-TCR	All TCR	Total
	(*n* = 147)	(*n* = 199)	(*n* = 346)
PD—*n* (%)	16 (14)	53 (33)	69 (25)
SD—*n* (%)	27 (24)	35 (22)	62 (23)
MR—*n* (%)	3 (3)	8 (5)	11 (4)
PR—*n* (%)	24 (21)	36 (22)	60 (22)
VGPR—*n* (%)	32 (28)	21 (13)	53 (19)
CR/sCR—*n* (%)	10 (9)	8 (5)	18 (7)
ORR—*n* (%)	66 (58)	65 (40)	131 (48)
Not evaluable—*n*	34	38	73

*PD* Progressive disease, *SD* stable disease, *MR* minimal response, *PR* partial response, *VGPR* very good partial response, *CR/sCR* complete response or stringent complete response, *ORR* overall response rate.

Of the 346 patients with MM relapsing on the index regimen and treated with a SoC subsequent line of therapy, 83 (83/346, 24%) were ≥75 years at relapse, and 263 (263/346, 76%) were <75 years. Among patients ≥75 years, the ORR to subsequent line of therapy was 38% (101/263), the median PFS was 4.4 (95% CI 3.5–6.3) months, and the median OS was 9.2 (95% CI 7.3–13.8) months. Among patients <75 years, the ORR to subsequent line of therapy was 36% (30/83), the median PFS was 4.1 (95% CI 3.4–5.1) months, and the median OS was 14.2 (95% CI 11.1–18.3) months.

### Outcomes among patients with TCR versus non-TCR MM

One hundred and ninety-nine (58% of the included study cohort, 199/346) patients had TCR MM and were treated with SoC regimens after progression on anti-CD38 mAb. Next line of therapy among patients with TCR MM was most commonly a combination of PI/steroid (53/199, 27%), IMiD/alkylator (46/199, 23%), PI/alkylator (29/199, 15%), or PI/IMiD (24/199, 12%). No patients received anti-BCMA therapy, an XPO1 inhibitor, or venetoclax directly after progression on the index regimen as these were not available as SoC in Canada. The ORR was 40% (65/161) to the first subsequent line of therapy, with 5% (8/161) of patients achieving a CR or better (see Table 3). The ORR of patients with TCR MM stratified by type of subsequent SoC regimen received is summarized in Table S3. Fourteen (14/199, 7%) patients with TCR MM were re-treated with an anti-CD38 mAb in combination with either an IMiD or PI in next line of therapy after progression on the index regimen, and 8 patients achieved at least a partial response (ORR 57%, 8/14 patients). Of the 147 patients who had non-TCR MM at progression on the index anti-CD38 mAb containing regimen, 123 (123/147, 84%) were had TCE MM.

Among patients with TCR MM, the median follow-up from the date of initiation of subsequent SoC therapy was 7.2 months. The median PFS from the start of subsequent therapy was 4.4 (95% CI 3.6–5.3) months, and the median OS was 10.5 (95% CI 8.5–13.8) months. We then performed sensitivity analyses among the patients with TCR MM to identify whether there was a subgroup that had inferior outcomes. Among the 48 patients with TCR MM and high-risk cytogenetic markers at diagnosis (48/199, 24%), the median PFS and OS from initiation of subsequent therapy after progression on the index regimen were 2.8 (95% CI 2.3–6.1) months and 8.5 (95% CI 4.7–12.4) months, respectively. Similarly, patients with TCR MM and a duration of response on the index anti-CD38 mAb containing regimen of less than 1 year had a trend towards a shorter PFS (161/199 [81%], median PFS 3.9 [95% CI 3.0–5.1] months) compared to patients with a response of ≥1 year (38/199 [19%], median PFS 6.1 [95% CI 3.9–7.3] months).

We compared the median PFS and OS from the start of the subsequent therapy for patients with non-TCR versus TCR MM when progressing on the index regimen. Both the median PFS and OS were significantly longer among patients with non-TCR versus TCR MM (median PFS 6.0 versus 4.4 months, respectively, *p* = 0.009; median OS 17.5 versus 10.5 months, respectively, *p* = 0.003 (see Fig. 3). Similarly, we compared the median PFS and OS from the start of the subsequent therapy for patients with TCR versus TCE (and not TCR) MM. The median PFS of patients with TCR vs TCE MM was similar (mPFS 4.4 (95% CI 3.6–5.3) months versus 4.5 (95% CI 3.9–7.9) months, *p* = 0.06), however OS was significantly shorter among patients with TCR vs TCE MM (mOS 10.5 (95% CI 8.3–13.4) months versus 17.4 (95% CI 12.3–26.6) months, respectively, p = 0.01), see Figure S2.

Outcomes of patients with TCR MM treated with a second subsequent SoC regimen (including compassionate belantamab mafodotin (*n* = 1) and selinexor (*n* = 5)) after anti-CD38 mAb progression were even poorer (*n* = 50 TCR patients, ORR 30% [15/50 patients], CR 2% [1/50 patients]; median PFS and OS from start of second subsequent SoC treatment were 2.8 (95% CI1.8–4.6) months versus 6.4 (95% CI 3.7–18) months, respectively).

### Predictors of response and outcomes

Multivariable regression found no significant association between age at initiation of subsequent therapy, sex, high-risk cytogenetics at diagnosis, the number of prior treatment lines, or the time from diagnosis to progression on the index regimen and PFS (Fig. S3). Patients achieving at least a VGPR on the index anti-CD38 mAb-containing regimen had a significantly longer PFS (HR 0.23, 95% CI 0.13–0.40, *p* < 0.001) and OS (HR 0.30, 95% CI 0.15–0.58, *p* < 0.001) after adjusting for confounders. When assessing factors associated with OS from the start of subsequent SoC therapy in a multivariable analysis, not surprisingly, younger patients had an improved survival compared to older patients after adjusting for confounders (HR 0.62, 95% CI 0.42–0.93, *p* = 0.02). Patients with a more aggressive disease course whose time from diagnosis to progression on the index regimen was shorter (<4 years) had a worse overall survival compared to patients with >4 years from diagnosis to progression (HR 1.59, 95% CI 1.09–2.36, *p* = 0.020), after adjusting for confounding. There was no association between sex, high-risk status at diagnosis, depth of response on the index regimen (summarized in Fig. S4).

## Discussion

In this large, real-world, retrospective, multi-center cohort study, we demonstrate that patients with MM relapsing on anti-CD38 mAb-containing regimens had poor outcomes when treated with SoC therapies, with a median PFS and OS of 4.6 months and 13.3 months from initiation of subsequent SoC treatment, respectively. Only 38% of patients had a partial response to SoC therapy after progressing on an anti-CD38 mAb. Outcomes of patients with TCR MM were dismal, with a median PFS of 4.4 months and median OS of 10.5 months from initiation of subsequent SoC treatment post anti-CD38 mAb progression. Importantly, there was a significant amount of attrition, with 22% of patients pursuing palliative care after relapsing on the index anti-CD38 mAb regimen.

Our cohort study reinforces findings of prior real-world studies reporting outcomes of patients with relapsed MM. In the retrospective MAMMOTH multi-center American study of patients with MM refractory to anti-CD38 mAbs who had received a median of 4 prior lines of therapy before the index regimen, the ORR was 31% and median PFS from initiation of subsequent therapy was 3.4, while the median OS from anti-CD38 mAb progression was 9.3 months [9]. The LocoMMotion trial was a prospective cohort study of patients with relapsed TCE MM treated with SoC regimens. It included 248 patients (90% of whom were treated in Europe) receiving 92 unique SoC treatment regimens at relapse, indicating the vast heterogeneity in treatment practices at disease relapse [14]. Patients had received a median of 4 prior lines of therapy and 92% had MM refractory to any anti-CD38 mAbs; the ORR was 29.8% with a median PFS and OS of 4.6 months and 12.4 months, respectively. Our study reports outcomes of the largest real-world cohort of patients with TCR MM to receive non-investigational, SoC therapy [9, 14–17]. However, despite differences in available treatments at relapse in various countries with different health-care systems and SoC drug accessibility, real-world studies of TCR MM consistently show a median OS of ≤12 months from the time of TCR status (as summarized in Table S4).

Findings from this study will be important to contextualize improvements in outcomes with newer therapies and will allow physicians to counsel patients about expected outcomes at relapse. We acknowledge that while this study is an indirect comparator for single-arm interventional studies evaluating newer agents, such comparisons need to be made cautiously. There are inherent differences in baseline fitness, comorbidities, organ function, and disease behavior that often lead to significant differences in outcomes of real-world versus clinical-trial patient cohorts treated with the same regimen [18, 19]. Unfortunately, given the retrospective nature of this study and the lack of consistent documentation regarding baseline fitness and comorbidities, these data were not included. Furthermore, while the intent of this study was to provide a real-world benchmark to compare efficacy outcomes of newer therapies, we recognize efficacy is not the only important metric to measure prior to incorporation of novel therapies into real-world practice. Multiple studies have shown patients with MM have increased comorbidities and healthcare resource utilization, and decreased quality of life over the course of their disease [17, 20, 21]. Therefore, further evaluation on the impact of treatment on quality of life, healthcare resource utilization, safety, and cost effectiveness will need to be considered when comparing standard of care versus newer treatments in future studies.

Our study was limited in that we were unable to identify characteristics associated with improved PFS or ORR with subsequent treatment, likely due to limited sample size and missing data. We were also not able to accurately determine outcomes among patients with high-risk cytogenetics because repeat FISH testing at relapse is often not reimbursed in our public-payer healthcare system. Moreover, there was variability in the FISH cytogenetic panels used at different sites during the period of study. However, a report of patients with TCR MM from Mayo Clinic showed that among the 44% of patients with FISH testing repeated at the time of TCR status, 87% were high-risk (defined at the presence of t(4;14), t(14;16), t(14;20), del(17p), TP53 mutation, and gain(1q)). Importantly, among patients with paired baseline and relapsed FISH data, 31% acquired chromosome 1q duplication and 25% acquired del(17p) at the time of TCR status. While the majority of patients had high-risk genetic markers at baseline, there was a notable increase in secondary cytogenetic abnormalities at relapse. This finding reinforces that baseline cytogenetic risk classification is not always representative of cytogenetic risk status at relapse. Therefore, comparison of outcomes of standard- and high-risk patients in clinical trials (where risk status is often re-evaluated at trial enrollment) versus our real-world cohort (where cytogenetic risk was assessed only at baseline), will need to be interpreted with caution. Furthermore, in Canada cancer treatment is reimbursed based on standardized provincial therapy funding guidelines built on national health technology assessment body recommendations (CADTH/INESS). Though anti-CD38 mAbs are increasingly being incorporated into the upfront treatment of patients with transplant-eligible or -ineligible MM [22–25], these agents were not publicly reimbursed in the front-line setting in Canada during the study period. Therefore, it is unclear whether the poor outcomes after progression on an anti-CD38 mAb will be generalizable to patients receiving these agents in the upfront treatment setting. Especially with the increasing use of quadruplet therapies, this possibility will need to be monitored closely.

In conclusion, this study provides a real-world benchmark of the most common therapies used to treat Canadian patients with relapsed or refractory MM. We show that patients with MM refractory to anti-CD38 mAbs, particularly TCR MM, have poor outcomes when treated with SoC regimens. Our observations are consistent with other publications reflective of other jurisdictions with a potentially different therapeutic landscape. This study highlights the ongoing unmet need for the development of and access to effective therapeutics for our patients.

## Supplementary information

Supplementary appendix

## Data Availability

The datasets generated during and/or analyzed during the current study are not publicly available due to privacy laws but access is available through the corresponding author on reasonable request. For original data please contact donna.reece@uhn.ca.
