# Supplementary material for: Real world data on outcomes of anti-CD38 antibody treated, including triple class refractory, patients with multiple myeloma: a multi-institutional report from the Canadian Myeloma Research Group (CMRG) Database
Source: Blood Cancer J. 2023 Dec 8;13(1):181. doi: 10.1038/s41408-023-00946-z (PMC10709576; doi:10.1038/s41408-023-00946-z)
Supplement: Supplementary file 1 — Supplementary appendix [file 41408_2023_946_MOESM1_ESM.docx]

**Supplementary Appendix:**

| **Table S1. Breakdown of standard of care treatment regimens used as subsequent therapy post CD38 mAb progression (total n=346)** | |
| --- | --- |
| **Regimen^a^** | **n (%)** |
| **PI only** | **112 (32)*** |
| Carfilzomib | 105 (30.3) |
| Bortezomib | 4 (1.2) |
| Ixazomib | 3 (0.9) |
| **IMID only** | **30 (9)*** |
| Pomalidomide | 23 (6.6) |
| Lenalidomide | 7 (2) |
| **Alkylator alone** | **4 (1)** |
| Cyclophosphamide | 3 (0.9) |
| Melphalan | 1 (0.3) |
| **IMID + PI** | **31 (9)** |
| Pomalidomide + Carfilzomib | 10 (2.9) |
| Pomalidomide + Ixazomib | 9 (2.6) |
| Pomalidomide + Bortezomib | 8 (2.3) |
| Lenalidomide + Carfilzomib | 2 (0.6) |
| Lenalidomide + Ixazomib | 2 (0.6) |
| **IMID + Alkylator** | **67 (19)** |
| Pomalidomide + Cyclophosphamide | 67 (19.4) |
| **PI + Alkylator** | **59 (17)** |
| Carfilzomib + Cyclophosphamide | 33 (9.5) |
| Bortezomib + Cyclophosphamide | 15 (4.3) |
| Ixazomib + Cyclophosphamide | 11 (3.2) |
| **IMID + PI + Alkylator** | **4 (1)** |
| Pomalidomide + Carfilzomib + Cyclophosphamide | 2 (0.6) |
| Pomalidomide + Ixazomib + Cyclophosphamide | 1 (0.3) |
| Pomalidomide + Bortezomib + Cyclophosphamide | 1 (0.3) |
| **CD38 mAb + Other** | **27 (8)** |
| Daratumumab + Pomalidomide | 10 (2.9) |
| Isatuximab + Pomalidomide | 5 (1.4) |
| Daratumumab + Cyclophosphamide | 3 (0.9) |
| Daratumumab + Ixazomib + Lenalidomide | 2 (0.6) |
| Daratumumab + Ixazomib + Cyclophosphamide | 2 (0.6) |
| Daratumumab + Ixazomib + Pomalidomide | 2 (0.6) |
| Daratumumab alone | 1 (0.3) |
| Daratumumab + Carfilzomib + Pomalidomide | 1 (0.3) |
| Daratumumab + Bortezomib | 1 (0.3) |
| **Other** | **12 (3)** |
| DPACE | 7 (2) |
| Steroid only | 3 (0.9) |
| DCEP | 1 (0.3) |
| KCD - ASCT - EloPd - Daratumumab maintenance | 1 (0.3) |
| ^a^A steroid (either prednisone or dexamethasone) was included with each regimen | |
| Abbreviations: proteosome inhibitor (PI); immunomodulatory drug (IMID); monoclonal antibody (mAb); dexamethasone & cisplatin & doxorubicin & cyclophosphamide & etoposide (D-PACE); dexamethasone & cyclophosphamide & etoposide & cisplatin (D-CEP); carfilzomib & cyclophosphamide & dexamethasone (KCD); elotuzumab & pomalidomide & dexamethasone (EloPd) | |

| **Table S2. Best response (among evaluable patients) to subsequent SoC therapy after progression on the index regimen among patients with high-risk MM at diagnosis** | | |
| --- | --- | --- |
|  | **High-risk MM** |  |
|  | **(n=85)** |  |
| PD – n (%) | 19 (28) |  |
| SD – n (%) | 18 (27) |  |
| MR – n (%) | 1 (2) |  |
| PR – n (%) | 16 (24) |  |
| VGPR – n (%) | 11 (16) |  |
| CR/sCR – n (%) | 3 (4) |  |
| ORR – n (%) | 30 (44) |  |
| Not evaluable - n | 17 |  |
| High risk cytogenetics was defined as the presence of t(4;14), t(14;16), or del(17p)  Abbreviations: Progressive disease (PD), stable disease (SD), minimal response (MR), partial response (PR), very good partial response (VGPR), complete response or stringent complete response (CR/sCR), overall response rate (ORR) | | |

**Table S3.** Triple class refractory (TCR) patient characteristics and outcomes, stratified by the type of standard of care regimen (non-clinical trial) received directly after progression on the index anti-CD38 monoclonal antibody containing regimen.

|  | **anti-CD38 + PI/IMiD** | **PI +**  **IMiD** | **PI +**  **steroid** | **PI +**  **Alkylator** | **IMiD + steroid** | **IMiD + Alkylator** | **Miscellaneous^a^** |
| --- | --- | --- | --- | --- | --- | --- | --- |
| **n** | **14** | **24** | **53** | **29** | **19** | **46** | **14** |
| High-Risk at diagnosis | 5 (36%) | 9 (38%) | 11 (21%) | 8 (28%) | 3 (16%) | 10 (22%) | 2 (14%) |
| Pomalidomide refractory | 6 (43%) | 8 (33%) | 7 (13%) | 7 (24%) | 1 (5%) | 3 (7%) | 10 (71%) |
| Carfilzomib refractory | 1 (7%) | 6 (25%) | 4 (8%) | 3 (10%) | 3 (16%) | 10 (22%) | 6 (43%) |
| Pomalidomide and carfilzomib refractory | 0 (0%) | 4 (17%) | 1 (2%) | 1 (3%) | 1 (5%) | 2 (4%) | 5 (36%) |
| Agent used – n(%) |  |  |  |  |  |  |  |
| Lenalidomide | 1 (7%) | 2 (8%) | - | - | 2 (11%) | 0 (0%) | 0 (0%) |
| Pomalidomide | 11 (79%) | 20 (83%) | - | - | 17 (89%) | 46 (100%) | 1 (7%) |
| Ixazomib | 5 (36%) | 8 (33%) | 1 (2%) | 5 (17%) | - | - | 0 (0%) |
| Bortezomib | 0 (0%) | 4 (17%) | 2 (4%) | 7 (24%) | - | - | 2 (14%) |
| Carfilzomib | 1 (7%) | 11 (46%) | 50 (94%) | 23 (79%) | - | - | 1 (7%) |
| Response rates – n(%) |  |  |  |  |  |  |  |
| PD | 3 (21%) | 9 (38%) | 14 (26%) | 6 (21%) | 3 (16%) | 10 (22%) | 8 (57%) |
| SD | 2 (14%) | 1 (4%) | 12 (23%) | 8 (28%) | 6 (32%) | 4 (9%) | 2 (14%) |
| MR | 2 (14%) | 2 (8%) | 0 (0%) | 0 (0%) | 1 (5%) | 2 (4%) | 1 (7%) |
| PR | 5 (36%) | 3 (13%) | 12 (23%) | 5 (17%) | 1 (5%) | 9 (20%) | 1 (7%) |
| VGPR | 0 (0%) | 2 (8%) | 6 (11%) | 4 (14%) | 2 (11%) | 6 (13%) | 1 (7%) |
| CR/nCR | 1 (7%) | 1 (4%) | 1 (2%) | 0 (0%) | 1 (5%) | 4 (9%) | 0 (0%) |
| Not evaluable | 1 (7%) | 6 (25%) | 8 (15%) | 6 (21%) | 5 (26%) | 11 (24%) | 1 (7%) |
| ORR | 46 % | 33% | 42% | 39% | 29% | 54% | 15% |
| Median PFS (months) | 7.5 (3.8-NR) | 3.3 (2.3-6.3) | 3.8 (2.5-5.4) | 5.5 (4.4-8.5) | 3.6 (2.7-8.7) | 6.1 (3.7-9.7) | 3.3 (1.3-NR) |
| Median OS (months) | 17.2 (10.6-NR) | 8.9 (4.5-NR) | 10.2(8.0-19.7) | 9.6 (7.4-NR) | 7.6 (4.4-NR) | 11.1 (8.2-23.5) | 7.3 (4.3-NR) |

^a^Miscellaneous treatments included alkylator/dexamethasone (n=2), steroid alone (n=3), daratumumab alone (n=1), therapy followed by salvage autologous stem cell transplant (n=2), bortexomib ± dexamethasone-cisplatin-doxorubicin-cyclophosphamide-etoposide (n=5), daratumumab + alkylator (n=1)
Abbreviations: proteosome inhibitor (PI), immunomodulatory drug (IMID), progressive disease (PD), stable disease (SD), minimal response (MR), partial response (PR), very good partial response (VGPR), complete response or stringent complete response (CR/sCR), overall response rate (ORR), overall survival (OS), progression free survival (PFS)

**Table S4. Summary of real-world studies reporting outcomes of patients with triple-class refractory multiple myeloma**

|  | **Total** (n) | **Received subsequent treatment** (n) | **Type of subsequent treatment** | Outcomes on subsequent therapy | | |
| --- | --- | --- | --- | --- | --- | --- |
|  |  |  |  | **mPFS** –  mo. (95% CI) | **mOS** –  mo. (95% CI) | **ORR** –  mo. (95% CI) |
| Gandhi *et al.*  Leukemia 2019  (**MAMMOTH**) (8) | 148 | - | SoC only (no selinexor, belamaf, idecel, melflufen) | - | 9.2 (7.1-11.2) | 29% |
| Bal *et al.* Leukemia 2022 (**MAMMOTH** **updated**) (13) | 177 | 177 | SoC only (no selinexor, belamaf, idecel, melflufen) | 2.8 (2.3-3.2) | 8.6 (6.8-10.3) | 30% |
| Mateos *et al.*  Leukemia 2022  (**LocoMMotion**) (12) | 183 | 183 | SoC, BCMA directed therapy (3%), Selinexor (<1%), venetoclax (2%) | 3.9 (3.4-4.6) | 11.1 (8.8-14.2) | 25% |
| Zanwar *et al.*  Leukemia 2022  (**Mayo Clinic**) (24) | 249 | 227 | SoC, CART (8%), BCMA BiTE/ADC (14%), selinexor (8%), venetoclax (16%) | 4 (3.5-5) | 12 (10.8-15.6) | 45% |
| Wang *et al.* Leukemia & Lymphoma 2022  (**US EHR database**) (14) | 221 | 173 | SoC (no selinexor, belamaf, idecel, melflufen) | 3.3 (2.7-3.9) | 11.6 (9.7-15.6) | - |
| Lee *et al.* CLML 2022  (**Connect MM Registry**) (15) | 232 | 155 | SoC; 3% received belamaf, selinexor, melflufen, or idecel | - | 10.8 | - |

Standard of care (SoC) was defined as therapies with proteosome inhibitors, immunomodulatory drugs, anthracyclines, alkylating agents, anti-CD38 monoclonal anitbodies. Abbreviations: Standard of care (SoC), belantamab mafodotin (belamaf), idecabtagene vicleucel (idecel), chimeric antigen receptor T-cell therapy (CART), overall survival (OS), progression free survival (PFS), overall response rate (ORR).


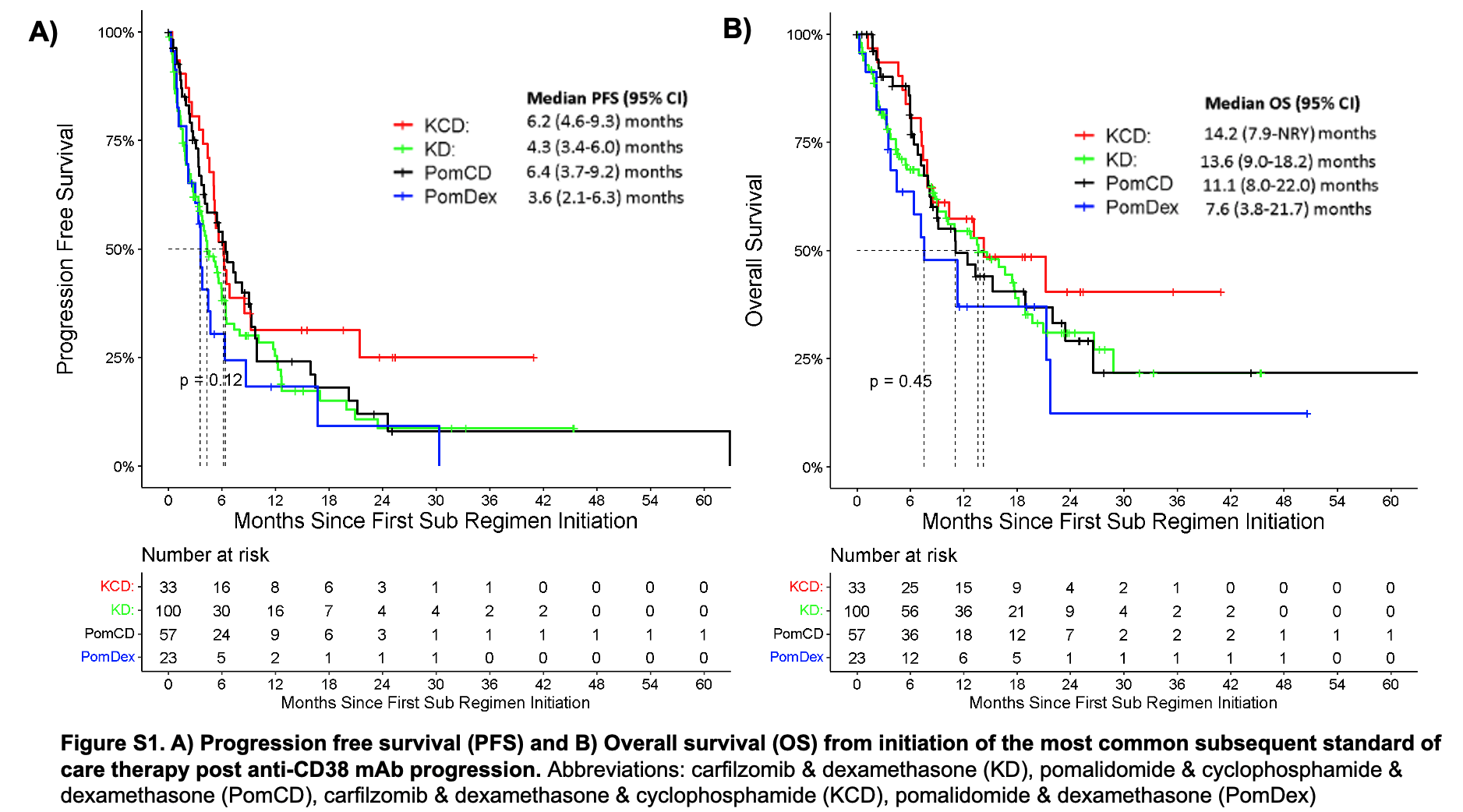


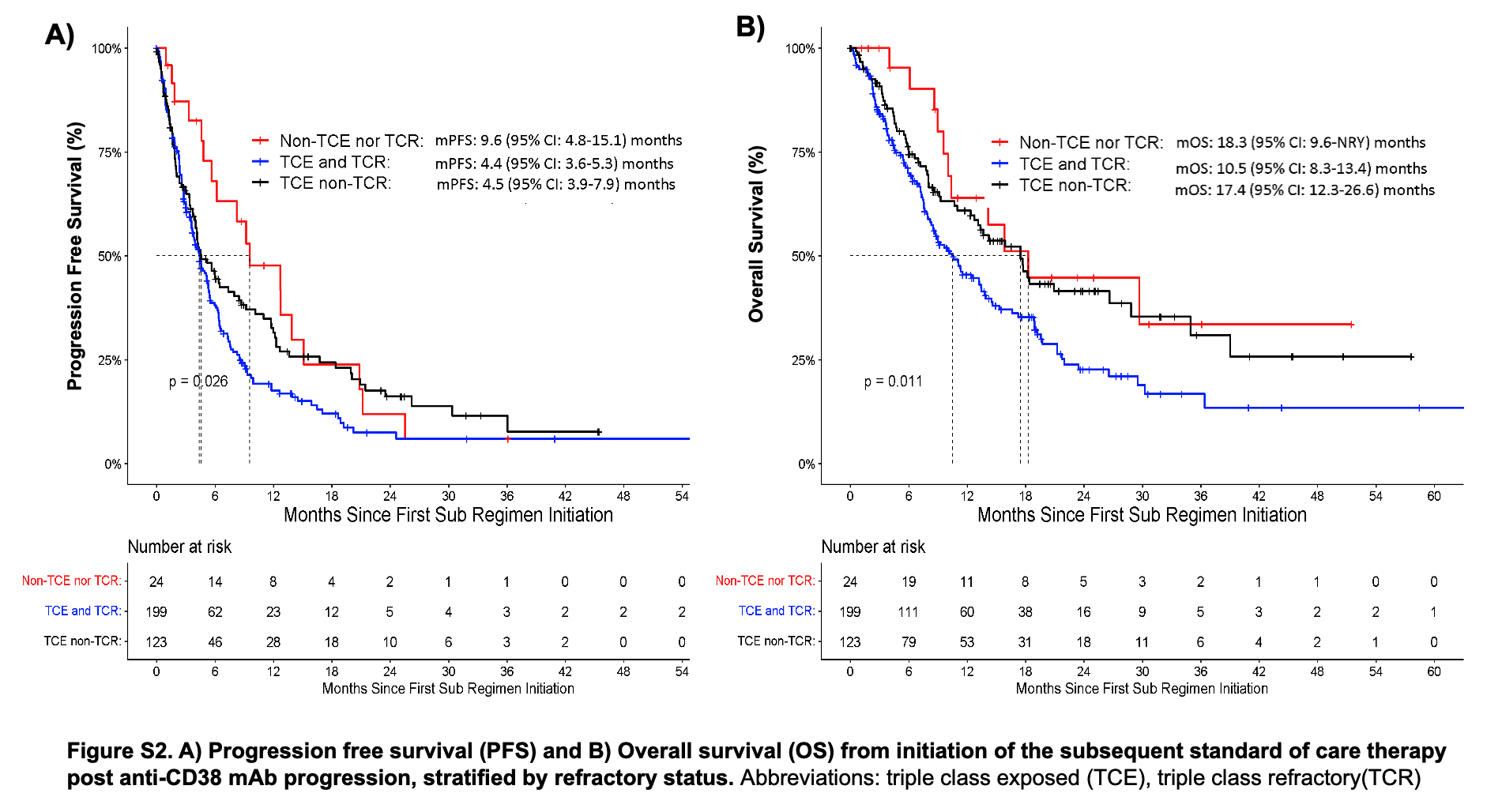


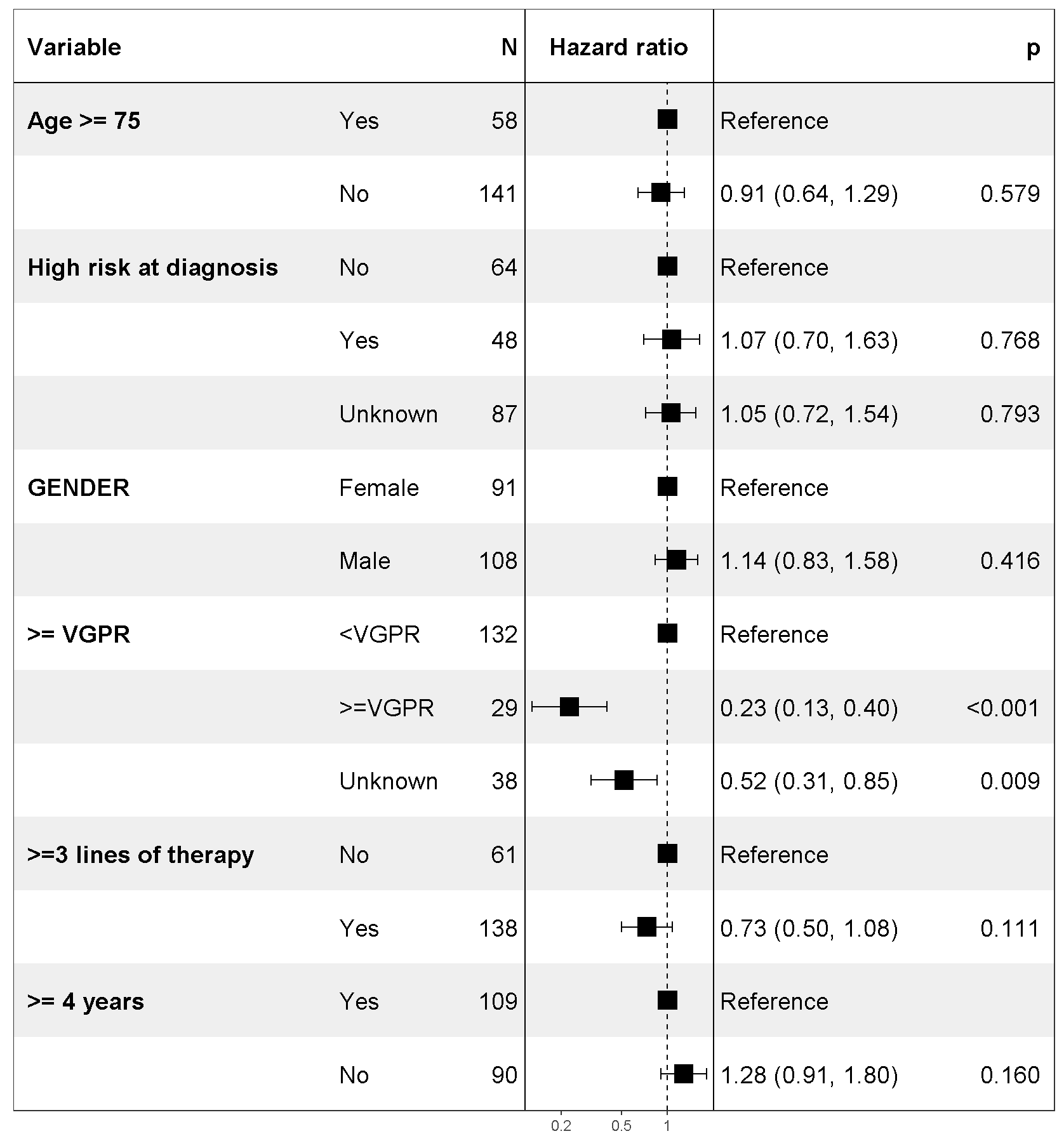


**Figure S3.** Forest plot summarizing the multivariable regression of factors associated with PFS from initiation of subsequent SoC therapy after relapse on the index regimen.


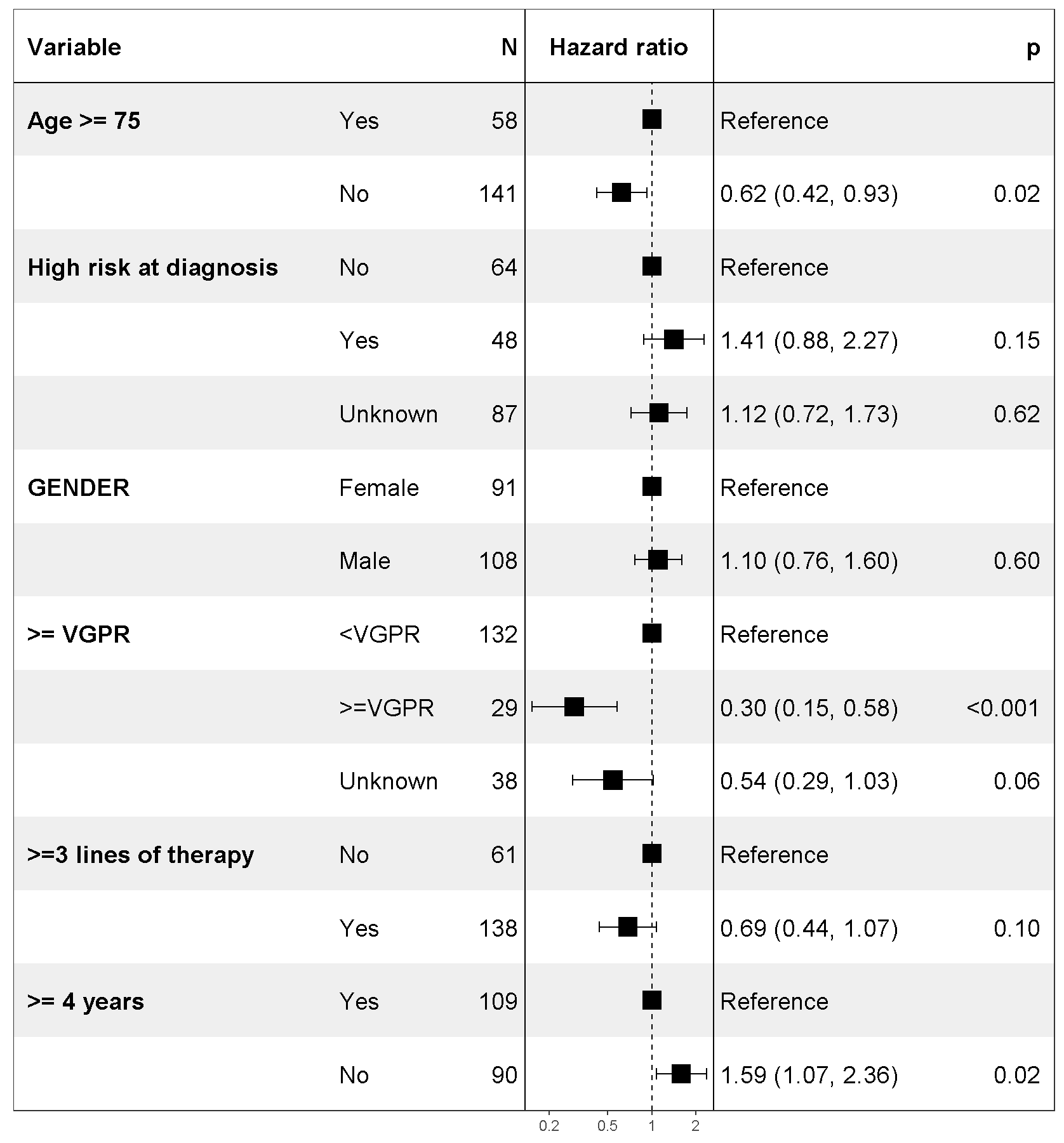


**Figure S4.** Forest plot summarizing the multivariable regression of factors associated with OS from initiation of subsequent SoC therapy after relapse on the index regimen.
